# Supplementary material for: Activation of the Anaphase Promoting Complex Restores Impaired Mitotic Progression and Chemosensitivity in Multiple Drug-Resistant Human Breast Cancer
Source: Cancers (Basel). 2024 Apr 30;16(9):1755. doi: 10.3390/cancers16091755 (PMC11083742; doi:10.3390/cancers16091755)

Supplemental Figure S1

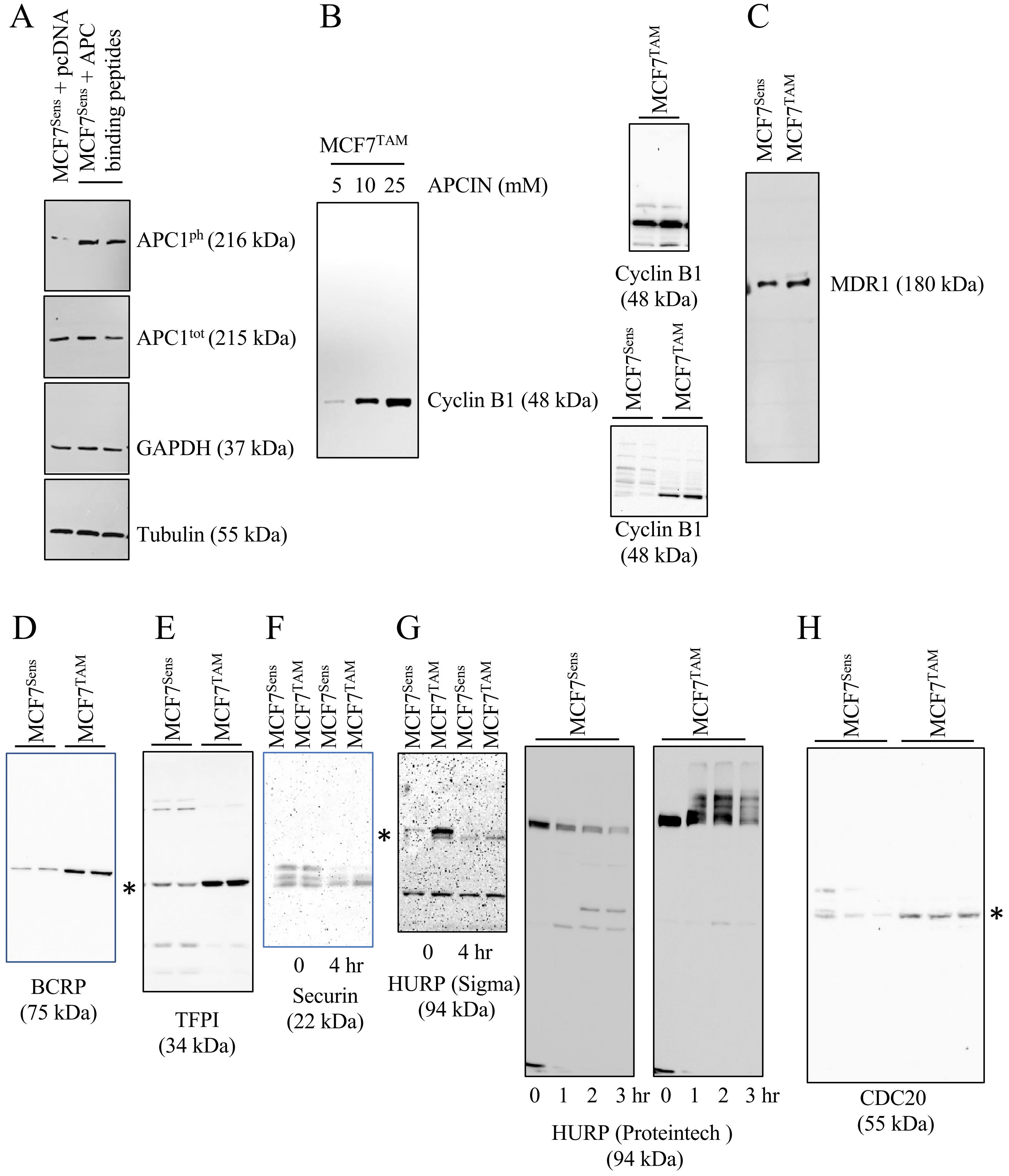

I

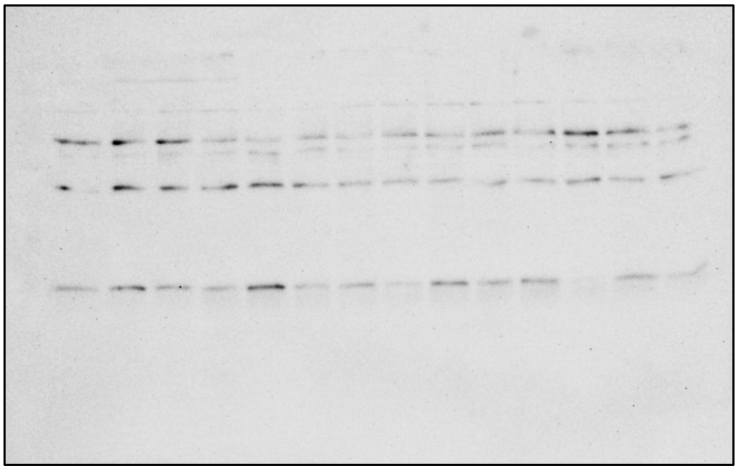

$H3^{Ser10phos}$ (15 kDa)

J

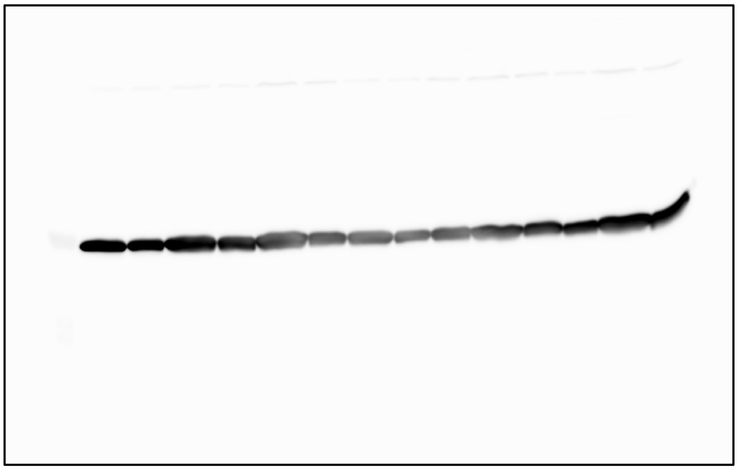

$H3^{tot}$ (15 kDa)

Supplemental Figure S2

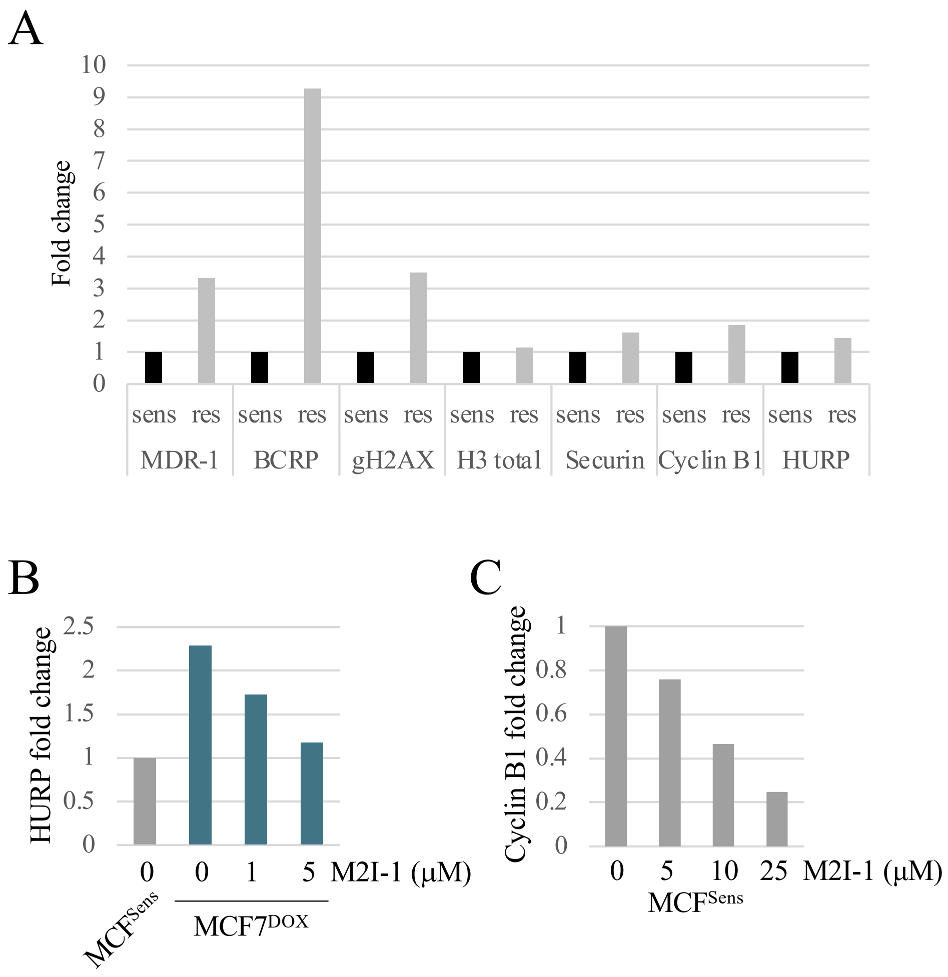

Supplemental Figure S3

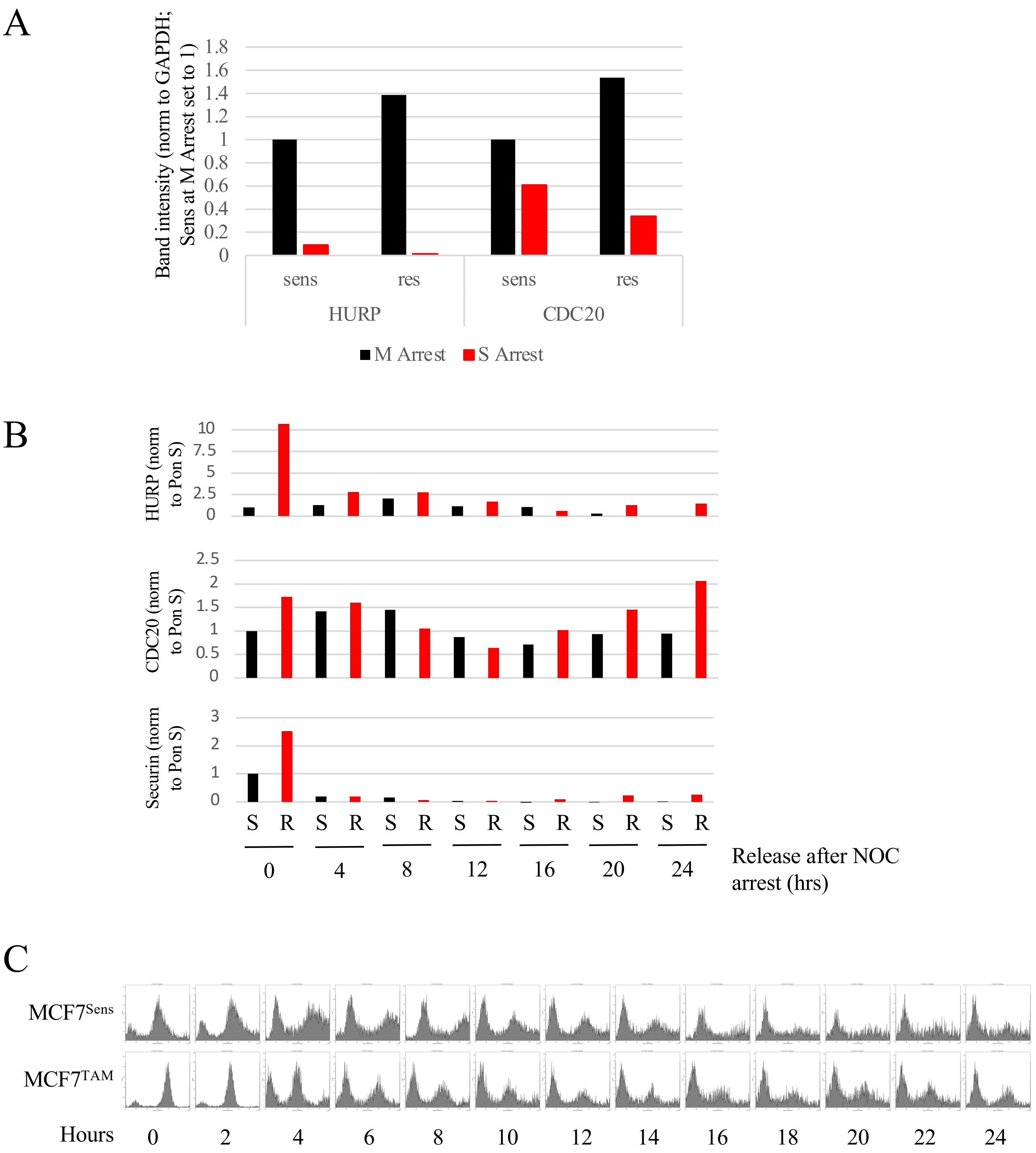

Supplemental Figure S4

A

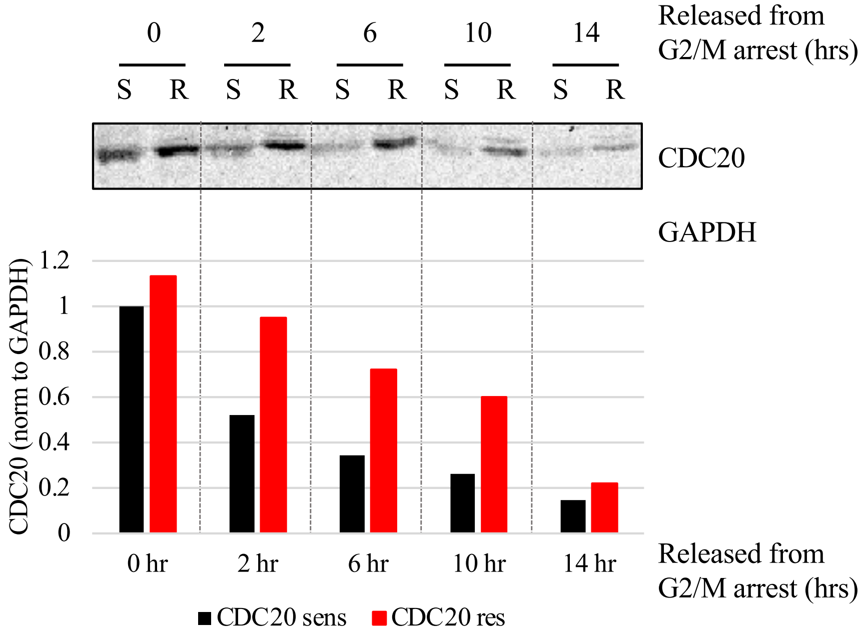

B

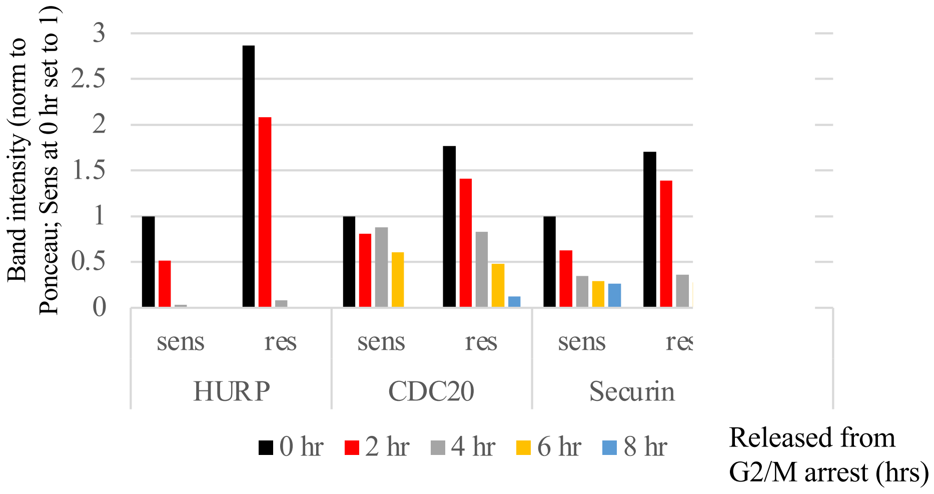

Supplemental Figure S5

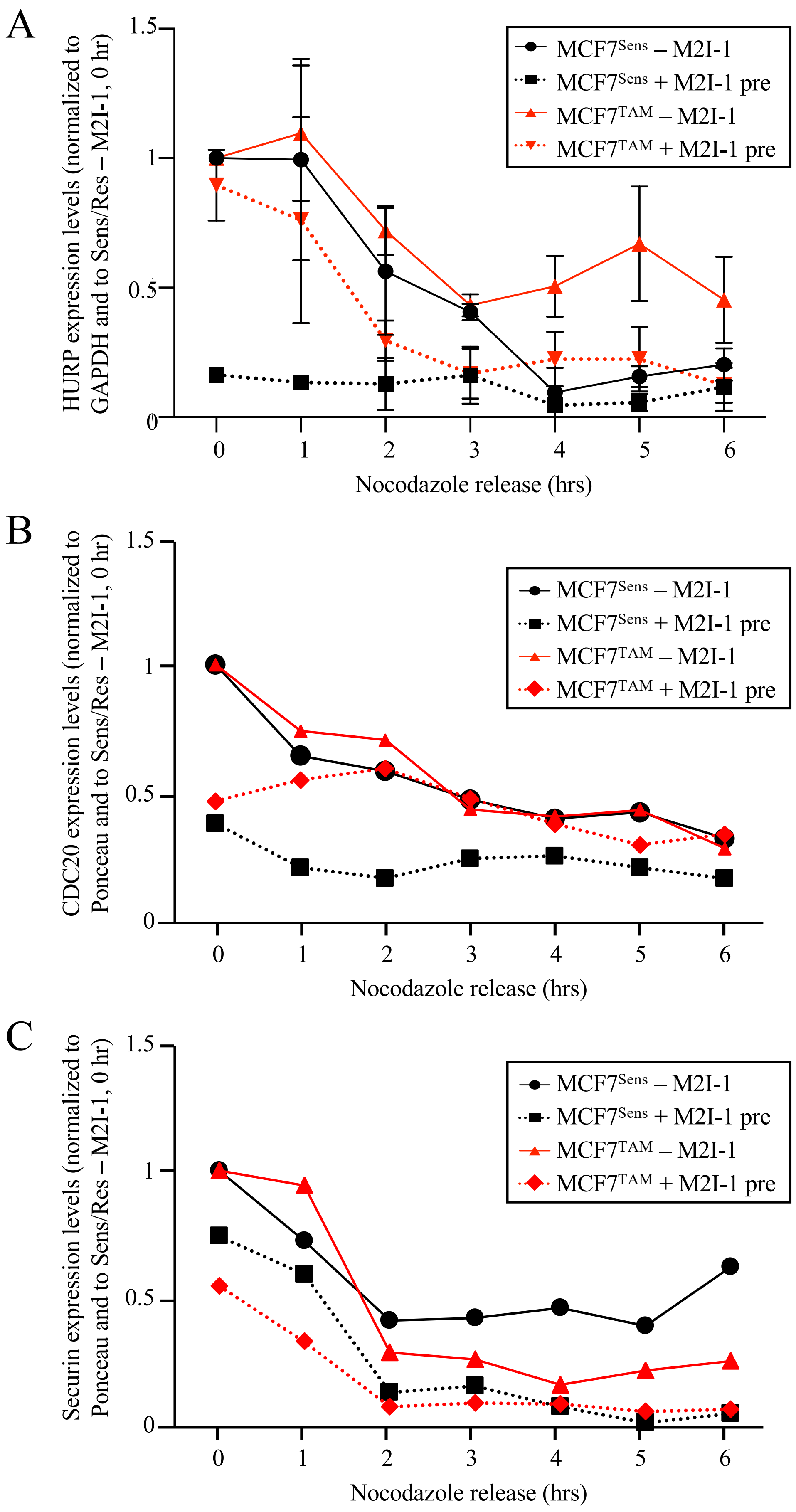

Supplement: Supplementary file 1 [file cancers-16-01755-s001.zip › cancers-2953880-supplementary.pdf]
